# A Simplified Surgical Approach for Distal Clavicle Non-union and Concurrent Skin Ulceration in an Older Adult: A Case Report

**DOI:** 10.7759/cureus.72557

**Published:** 2024-10-28

**Authors:** Sreenivasulu Metikala, Madana Mohana Reddy Vallem, Khalid Hasan

**Affiliations:** 1 Orthopaedics, Virginia Commonwealth University, Richmond, USA; 2 Orthopaedics, Virginia Commonwealth University School of Medicine, Richmond, USA

**Keywords:** distal end clavicle fractures, non-operative management, non-union fracture, open fracture, skin necrosis, skin ulcer

## Abstract

We present a case of asymptomatic non-union of distal clavicle fracture in an older adult who experienced late-onset skin dehiscence and a secondarily open fracture. We adopted a simplified surgical approach involving resection of the protruding medial clavicular fragment instead of fixation of the non-union. This approach led to the rapid healing of soft tissues while maintaining an ulcer-free status and adequate shoulder function at the one-year follow-up. Skin necrosis, a frequent complication of acute distal clavicle fractures, may also occur in non-union. In a low-demand elderly patient, a conservative surgical approach involving local non-union spur resection yielded favorable outcomes.

## Introduction

Bony non-union is a known complication and often entails a long and laborious treatment process [1]. Non-union is a common complication associated with displaced distal clavicle fractures and can be as high as up to 23% [2].

For elderly patients, conservative management is the preferred approach due to its minimal functional impact [2]. Skin tenting is another complication of distal clavicle fractures caused by the superior displacement of the medial clavicular fragment. This is typically seen in acute cases and requires surgical reduction and fixation to minimize the risk of subsequent skin necrosis and ulceration [3]. Notably, such a presentation has not been reported in a non-union situation.

We present a case of an elderly male patient with a non-union of the distal clavicle fracture, which led to delayed skin ulceration due to the prominent fracture fragment. His non-union, despite the residual displacement, remained well tolerated until the occurrence of overlying skin necrosis and ulceration. As is well known, operative reconstruction of distal clavicle fracture non-union in the elderly comes with its own risks and complications. Non-operative treatment is also unlikely to heal the ulcer due to the persistent mechanical pressure effects from the displaced non-union clavicular fragment. Our approach involved a simplified surgical procedure that entailed the excision of the ulcer and resection of the underlying non-union fracture spur. This strategy served as an effective offloading intervention and resulted in uneventful healing of the skin ulceration, avoiding the need for major operative internal fixation of his non-union and its potential complications. At the final one-year follow-up, the patient maintained an ulcer-free status and had preserved shoulder function.

## Case presentation

**Figure 1:**
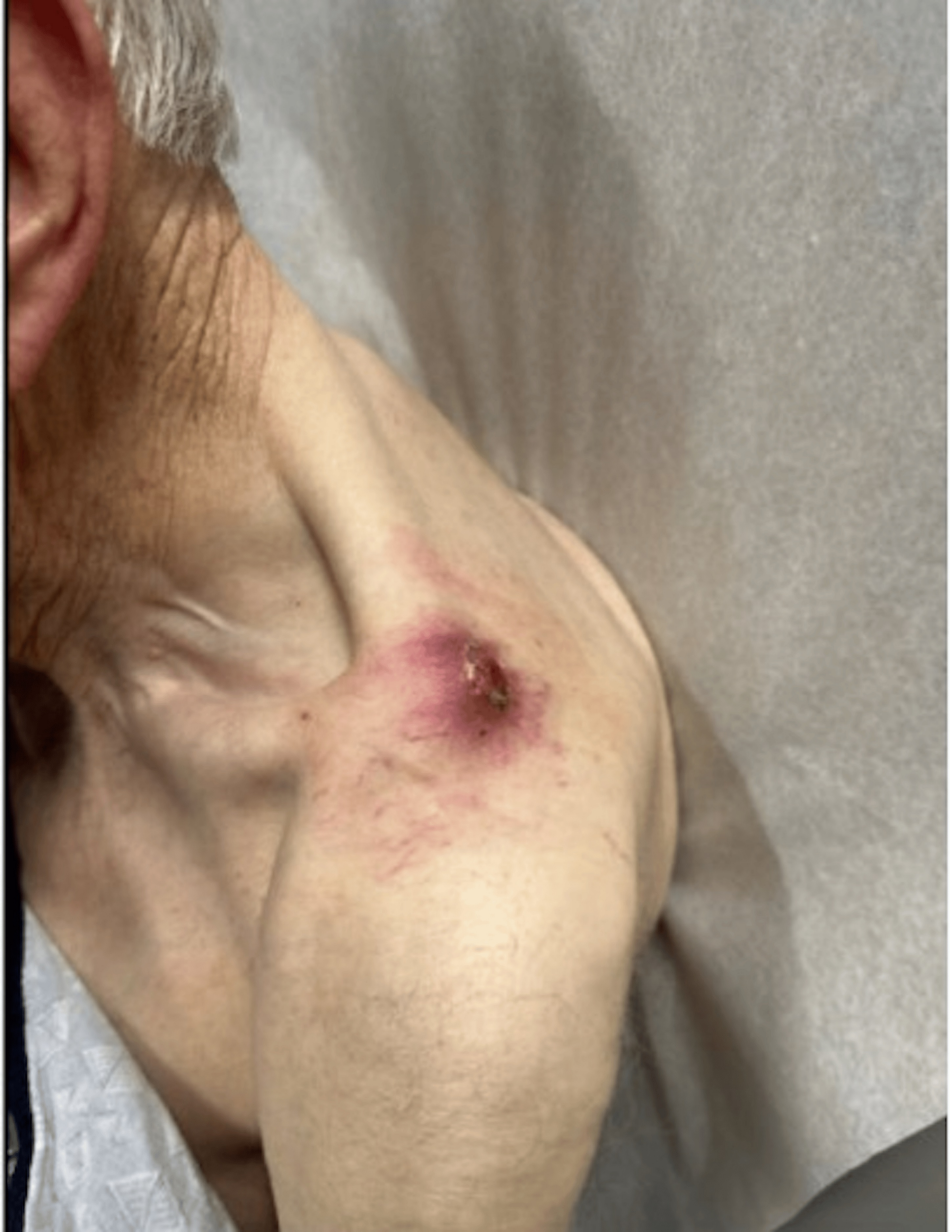
Clinical photograph of the left clavicle non-union site with overlying ulceration

A right-hand-dominant male patient in his 80s, status post-non-union left distal clavicle fracture, presented with localized skin dehiscence and pain of three-week duration. Upon clinical examination, a 3 x 2-cm ulcer with a hypertrophic granulating base was observed, accompanied by tenderness and thin serous drainage (Figure 1).

**Figure 2:**
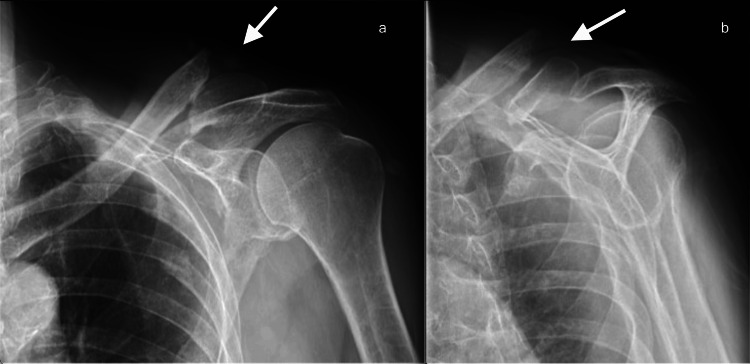
Radiographs of left shoulder: (a) anteroposterior (AP) and (b) scapular Y views showing distal clavicle non-union with superior displacement of the medial fragment

The prominent non-union fragment could be felt within the ulceration, surrounded by local erythema and warmth. Plain radiographs revealed distal clavicle fracture non-union with a prominent spike from the medial clavicle fragment at the soft tissue defect site (Figure 2). 

A Gram stain and culture of the draining material did not identify any microorganisms. Laboratory testing demonstrated normal white cell counts, erythrocyte sedimentation rates, and C-reactive protein levels. Upon reviewing the patient's medical records, it was noted that the initial injury occurred six months ago and was conservatively treated. As per the last follow-up note, three months post-injury, the patient reported resuming most activities despite the presence of non-union in the radiographs. The patient was doing well until he developed a 1-cm blister at the non-union site three weeks before the current visit. His family doctor prescribed oral antibiotics (cephalexin 500 mg for one week), but the lesion worsened, resulting in full-thickness skin ulceration. The patient was otherwise systemically well and had stable vitals. While the patient denied a re-injury, he did acknowledge wearing suspenders for his pants recently, which could have possibly caused direct external pressure on the prominent bone spike leading to the skin lesion. His past medical history includes coronary artery disease with prior triple bypass, hypertension, and asthma controlled on medication. At this point, local wound care was deemed inadequate due to the underlying protruding bone fragment. As a result, surgery was discussed, including non-union takedown with internal fixation versus local resection of prominent fracture spur. After reviewing the pros and cons of each option, the patient was recommended to undergo the latter option.

**Figure 3:**
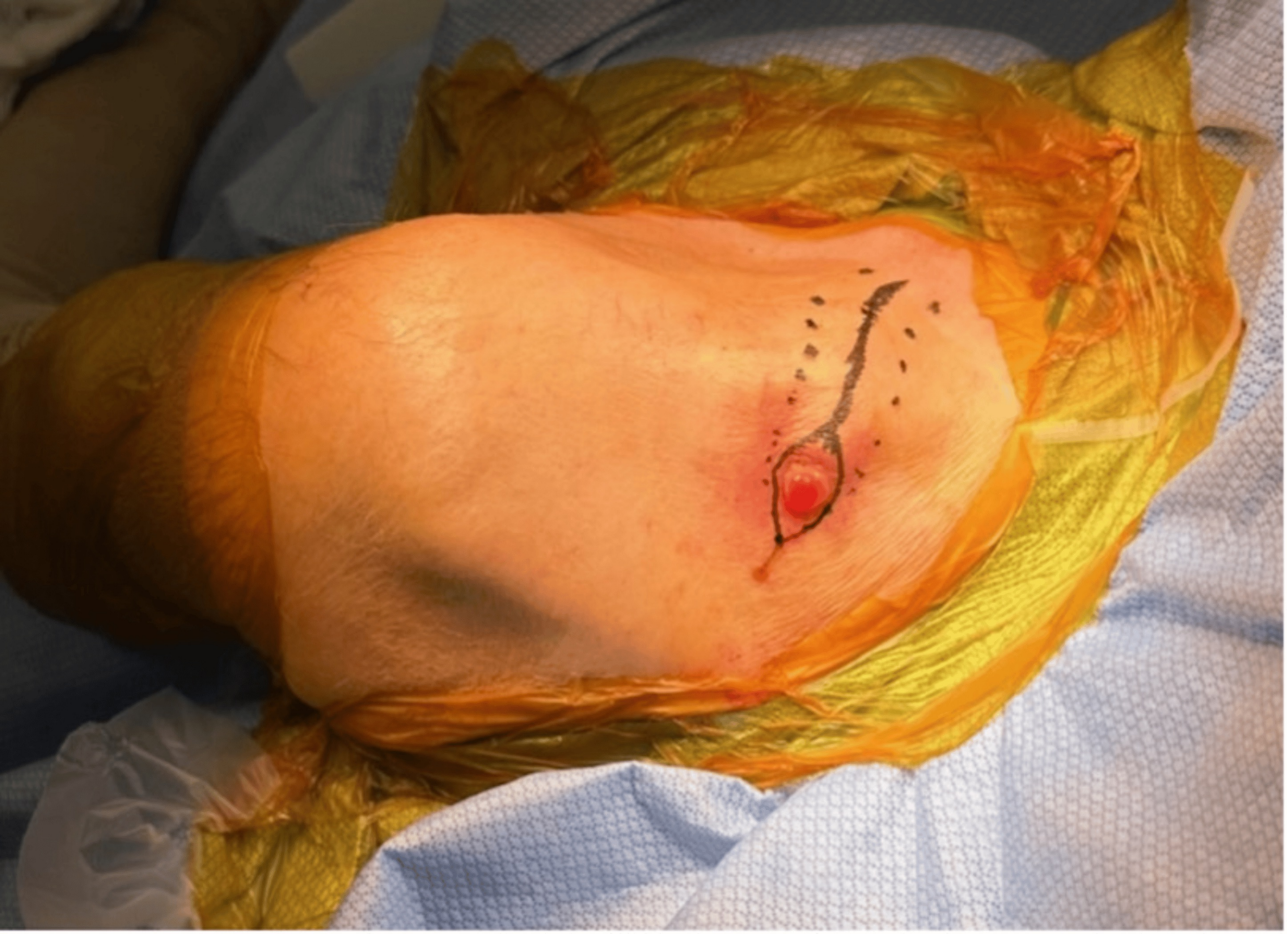
Clinical photograph showing the planned surgical incision

**Figure 4:**
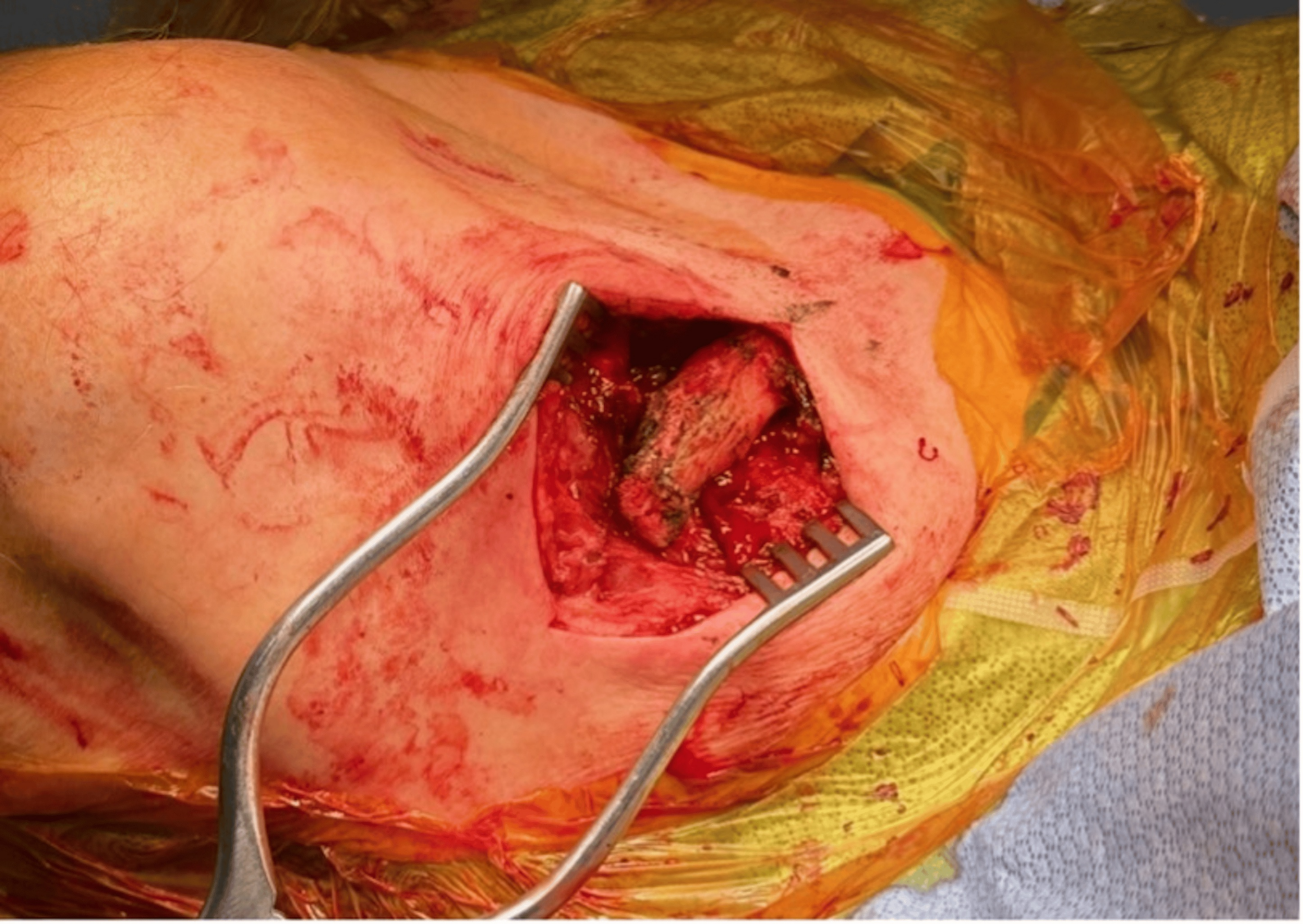
Intraoperative photograph showing non-union with a prominent medial clavicular fragment

The patient was placed supine on the operating table and was given general anesthesia. After sterile preparation (Figure 3), a 5-cm skin incision was made over the distal clavicle, excising the ulcer margins in an elliptical fashion. The non-union site was accessed by dissecting the subcutaneous tissues and platysma (Figure 4).

**Figure 5:**
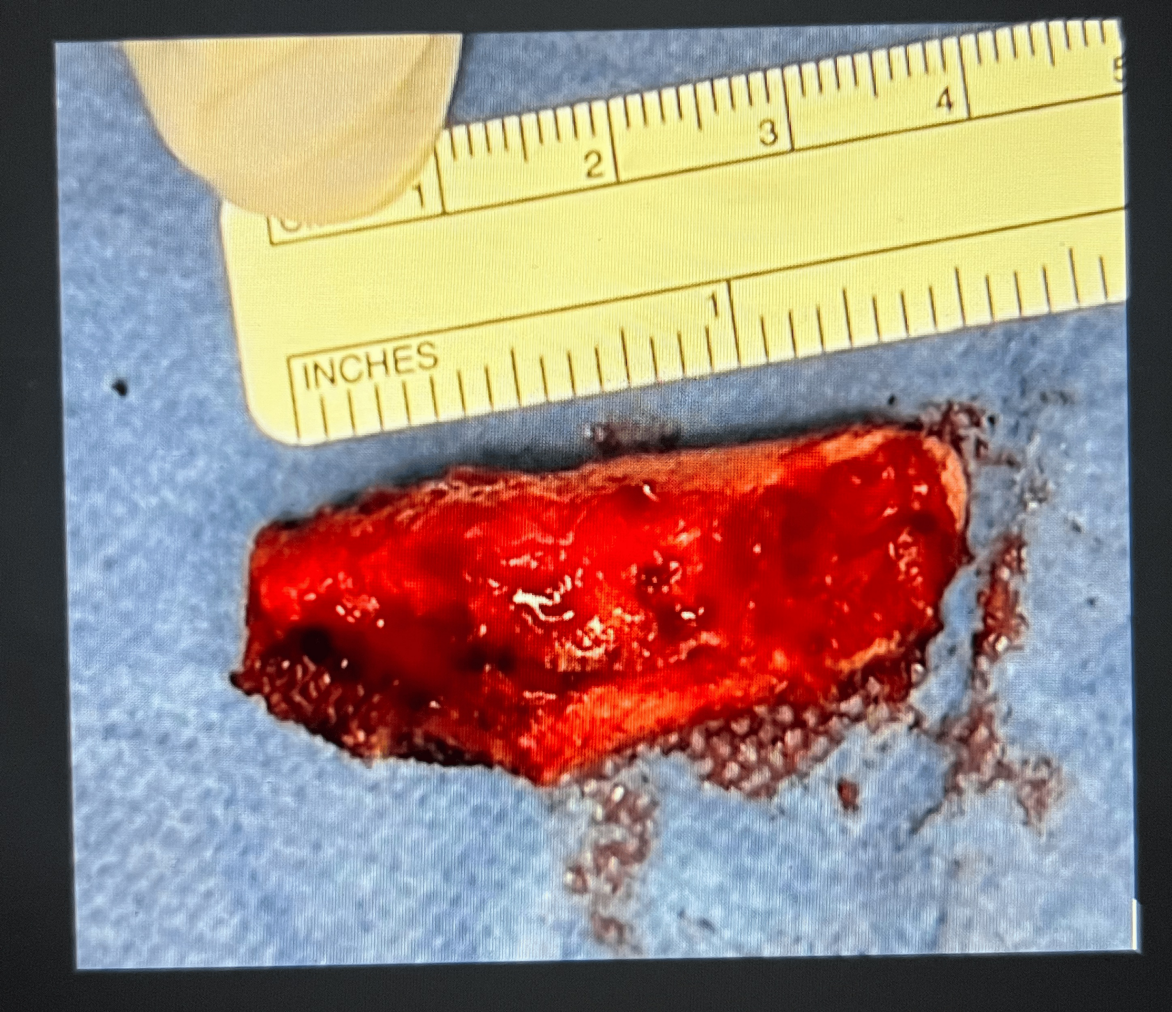
Excised non-union end of the medial clavicular fragment

**Figure 6:**
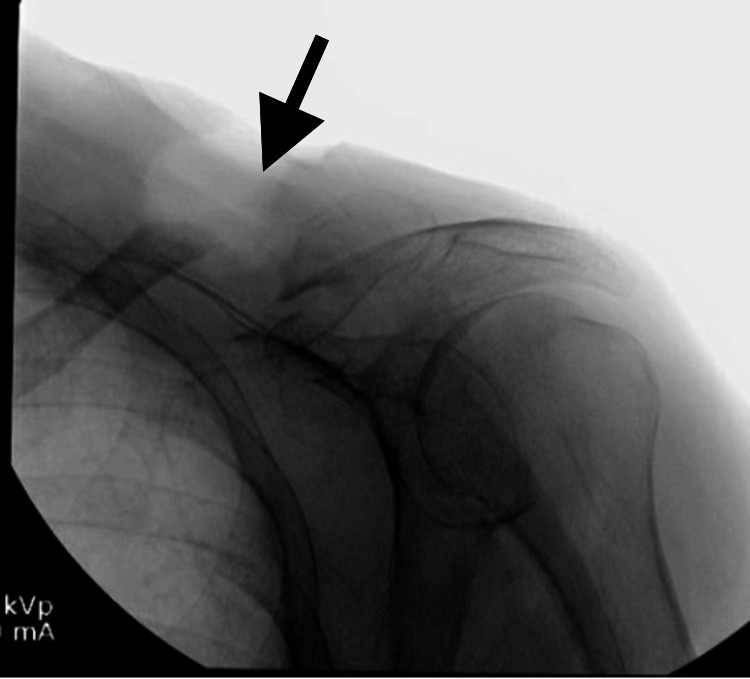
Intraoperative fluoroscopy image showing adequate excision of the non-union end of the medial fragment

No signs of pus or exudate deposits were noted in the deeper planes. After subperiosteal dissection, a 3-cm medial fragment was resected using a sagittal saw (Figure 5), and the edges were smoothed with a hand rasp. The excised bone, along with deep tissue samples, was sent to the microbiology department. Direct digital palpation and intraoperative fluoroscopy confirmed no residual bone spikes (Figure 6). 

The wound was then copiously irrigated with saline and closed in layers. The arm was placed in a sling, and the patient was discharged home the same day. The sling was discontinued after one week, and shoulder range of motion exercises were initiated. The sutures were removed on the 10th postoperative day, and primary healing of the incision was noted. Tissue and bone cultures showed no signs of microbial growth. After 12 weeks, the patient resumed all previous activities, including wearing pant suspenders, without further skin issues.

**Figure 7:**
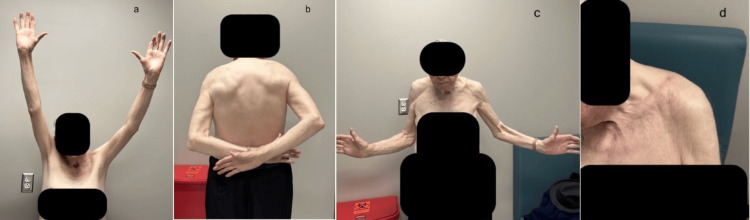
One-year postoperative clinical photographs showing near full range of motion in the left shoulder with healed surgical scar by primary intention: (a) abduction; (b) internal rotation; (c) external rotation; (d) healed scar

The final evaluation, conducted 18 months after the index injury and one year after the surgery, revealed a near-equal range of motion as well as strength in both shoulders (Figure 7). 

**Figure 8:**
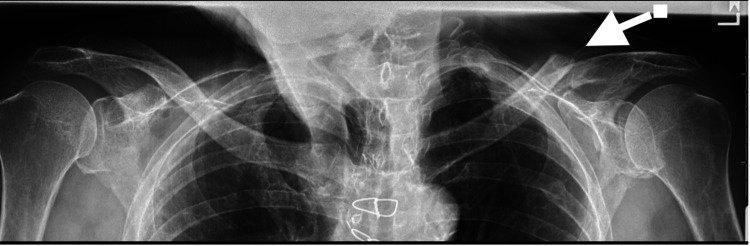
One-year postoperative radiograph anteroposterior view showing even alignment of both clavicles with no residual superior displacement of the left medial clavicular fragment

There was a 20-degree limitation of forward elevation in the left shoulder, while the overlying skin and soft tissues remained intact. The final radiographs demonstrated gap non-union of the distal clavicle but without any residual prominence of the fracture fragments (Figure 8).

## Discussion

A Neer type II distal clavicle fracture with displacement is an unstable fracture pattern and poses a management challenge. For young and active patients, primary open reduction and internal fixation (ORIF) are often recommended using various internal fixation techniques to minimize the risk of non-union [2,3]. Despite this, the functional impact of distal clavicle fracture non-union remains unclear. The results of a randomized controlled study by Hall et al. showed no difference in functional outcomes between groups treated operatively and non-operatively, although union rates were better in the operative group [4]. Another study of 101 patients with displaced fractures who underwent conservative treatment revealed that 21% developed asymptomatic non-union, while only 14% needed delayed surgical intervention [5]. Thus, primary non-operative management of distal clavicle fracture in an older adult, even when the fracture is displaced, is considered a valid option.

Another major complication of displaced distal clavicle fracture is the integrity of the soft tissue envelope. Visible deformity with skin tenting over the prominent edge of the medial clavicular fracture fragment is directly proportional to the amount of initial displacement. Persistent skin tenting leads to skin necrosis and ulceration, thereby converting a closed injury into an open fracture with subsequent risk of osteomyelitis. This impending open fracture pattern is usually seen in acute situations and requires prompt operative intervention [6-8]. To the best of the authors’ knowledge, there is no description of late-onset skin ulceration in a non-union of distal clavicle fracture that was otherwise asymptomatic. Perskin and Egol reported a closed type II fracture in an 84-year-old male patient that turned into an open fracture a month after the injury [9]. It was addressed by ORIF using a plate and screws along with coracoclavicular ligament reconstruction, but the hardware failed, and the patient had a recurrence of deformity after seven weeks.

In this case, the initial non-operative treatment led to asymptomatic non-union with satisfactory function. After six months, the patient experienced a violation of the soft tissue envelope due to repeated external shear forces from wearing suspenders. As a result, the patient's skin necrosed and eventually broke down, requiring prompt surgical treatment before a deep infection set in.

Treating non-union with the conventional method of ORIF using hardware, soft tissue allograft, and/or bone graft can pose unique risks and complications in elderly patients. Simple ulcer excision and local resection of the prominent medial fragment would be a more feasible approach. This approach effectively offloaded the ulcer and led to primary healing of the soft tissues with satisfactory outcomes. Such an approach, to the best of our knowledge, has not been reported before. We believe that surgeons can apply this approach and treat these fracture non-unions with good outcomes.

## Conclusions

Skin necrosis, a frequent complication in acute distal clavicle fractures with displacement, may also arise in a non-union situation. This poses a unique challenge in terms of treatment options. In an elderly and low-demand patient, a conservative surgical approach involving local resection of a prominent bone spur is likely to result in soft tissue healing and favorable outcomes, despite not performing operative fixation of non-union. Conventional fixation with hardware can be risky in this group of populations. We believe that simple excision of the non-union fragment can lead to satisfactory clinical and functional outcomes.
